# Clinical and imaging findings of discharged patients with SARS-CoV-2 positive anal swab samples: a descriptive study

**DOI:** 10.1186/s12879-020-05363-2

**Published:** 2020-09-01

**Authors:** Hui Zhou, Liping Zhu, Yueshuang Leng, Dongcui Wang, Harrison X. Bai, Zeng Xiong, Linbo Shi, Weihua Liao

**Affiliations:** 1 Department of Radiology, Xiangya Hospital, Central South University, Changsha, 410008 Hunan China; 2 Department of Radiology, Yongzhou Central Hospital, Yongzhou, 425006 Hunan China; 3 Department of Diagnostic Imaging, Rhode Island Hospital, Providence, Providence, RI 02903 USA; 4 Molecular Imaging Research Center of Central South University, Changsha, 410008 China

**Keywords:** COVID-19, Discharged patients, Anal swab, CT scan

## Abstract

**Background:**

To explore the clinical features and CT findings of clinically cured coronavirus disease 2019 (COVID-19) patients with viral RNA positive anal swab results after discharge.

**Methods:**

Forty-two patients with COVID-19 who were admitted to Yongzhou Central Hospital, Hunan, China, between January 20, 2020, and March 2, 2020, were tested for severe acute respiratory syndrome coronavirus 2 (SARS-CoV-2) using anal swab viral RT-PCR. In this report, we present the clinical characteristics and chest CT features of six patients with positive anal swab results and compare the clinical, laboratory, and CT findings between the positive and negative groups.

**Results:**

The anal swab positivity rate for SARS-CoV-2 RNA in discharged patients was 14.3% (6/42). All six patients were male. In the positive group, 40% of the patients (2/5) had a positive stool occult blood test (OBT), but none had diarrhea. The median duration of fever and major symptoms (except fever) in the positive patients was shorter than that of the negative patients (1 day vs. 6 days, 4.5 days vs. 10.5 days, respectively). The incidence of asymptomatic cases in the positive group (33.3%) was also higher than that of the negative group (5.6%). There were no significant differences in the CT manifestation or evolution of the pulmonary lesions between the two groups.

**Conclusion:**

In our case series, patients with viral RNA positive anal swabs did not exhibit gastrointestinal symptoms, and their main symptoms disappeared early. They had similar CT features to the negative patients, which may be easier to be ignored. A positive OBT may indicate gastrointestinal damage caused by SARS-CoV-2 infection.

## Keypoints

Discharged patients with an anal swab positive for SARS-CoV-2 RNA could be concealed and easily ignored. This positive result may not represent a recurrence of the disease, but rather represents the intermittent excretion of the virus.

## Background

COVID-19 is a novel acute respiratory infectious disease caused by severe acute respiratory syndrome coronavirus 2 (SARS-CoV-2) [1], which is highly contagious. As of August 3, 2020, 88,573 COVID-19 patients have been diagnosed in China. In addition, 17.9 million laboratory-confirmed cases and 686,703 deaths have been reported in 215 countries and regions outside of China, and the number of infected people is still rising rapidly [2–5].

As most patients with COVID-19 show symptoms of respiratory tract infections, the current diagnosis, and epidemic control standards for COVID-19 rely on the collection of throat swabs, sputum, and lower respiratory tract secretions for quantitative reverse transcription polymerase chain reaction (qRT-PCR) based detection of SARS-CoV-2 RNA. Patients can only be discharged after their RNA throat swab tests are negative twice (sampling interval of at least 1 day) [6]. Some researchers have found a small number of patients who present with SARS-CoV-2 RNA positive stool samples [7], and that the respiratory RNA test results may not be consistent with those from stool samples [8, 9]. However, the clinical and imaging findings from discharged patients with negative throat-swab but positive anal-swab RNA test results have not yet been reported.

In this study, we wanted to evaluate the clinical characteristics and radiographic features of patients with SARS-CoV-2 RNA positive anal swabs after discharge, with a view to improving our understanding of the pathology and risk profiles of these patients.

## Methods

### Study design and participants

This study was approved by the Ethics Commission of Xiangya Hospital Central South University (approval number 202002019), which waived the need for written informed consent due to the nature of COVID-19. All patients with a confirmed COVID-19 diagnosis admitted to our institution who met the national discharge criteria between January 20, 2020, and February 22, 2020, were enrolled in this study. All enrolled patients were diagnosed using the WHO interim guidance [10] and confirmed by a positive SARS-CoV-2 RNA throat swab qRT-PCR result. Patients were characterized as mild, common, severe, and fatal types based on the clinical guidelines for COVID-19 (Trial Version 5) proposed by China National Health Commission [6]. All confirmed cases were subject to clinical observation and treated in the isolation ward of our institution. The development of a treatment plan was based on the COVID-19 guidelines (Trial Version 5) [6]. Discharge criteria of patients with COVID-19 [6] were as follows: 1) body temperature dropped to normal for more than 3 days, 2) significant improvement in respiratory symptoms, 3) obvious inflammatory absorption on pulmonary imaging, and 4) negative RNA test results for two consecutive respiratory samples (sampling interval ≥ 1 day). Data on the clinical characteristics, laboratory findings, treatment, and outcomes were collected from electronic medical records.

Forty-two confirmed COVID-19 patients meeting the discharge criteria were subjected to anal swab sample collection and these samples were then tested for SARS-CoV-2 RNA. Throat and anal swab samples were collected and evaluated using the Chinese Center for Disease Control and Prevention–standardized quantitative polymerase chain reaction assay [11]. All samples were processed by the Department of Laboratory Medicine at Yongzhou Central Hospital. All enrolled patients were divided into positive (6 cases) and negative (36) groups based on their qRT-PCR results. Patients with positive anal-swab results were kept in isolation and placed under medical observation for a further 2 weeks at a secondary site to prevent spread and all the respiratory samples were rechecked to ensure that they were viral RNA negative. Anal swab samples were reevaluated 7 days later, with five patients returning a negative result. The two severe COVID-19 patients had a negative anal swab test 9 days later (Table 1). Patients were released only when we received two consecutive negative results (sampling interval ≥ 1 day) and at the same time, all their close contacts were found and quarantined at home for 14 days in accordance with the guidelines [6]. Thirty-one patients underwent a CT scan at admittance and all enrolled patients underwent a CT scan on discharge, with 25 patients needing another CT scan 7–14 days after discharge.

**Table 1:** Clinical Characteristics and CT findings of patients with positive anal swab viral RNA

	Patient 1	Patient 2	Patient 3	Patient 4	Patient 5	Patient 6
Sex	Male	Male	Male	Male	Male	Male
Age-ranges, y	15–20	5–10	60–70	40–50	20–30	30–40
Clinical type	Common	Common	Severe	Severe	Mild	Common
Maximum temperature, °C	36.9	36.0	36.1	39.5	37.5	37.8
Diarrhea	No	No	No	No	No	No
OBT		Negative	Positive	Positive	Negative	Negative
Duration of fever, d	0	0	0	5	2	10
Duration of major symptoms (except fever), d	0	5	7	4	0	5
Duration of throat swab positive, d	11	9	13	12	2	9
Duration of anal swab positive, d	7	7	9	9	7	7
Hospital stays, d	13	15	20	21	5	14
CT score on admission	0	1	17	13		
CT score on discharge	0	0	14	10	0	0
CT score after discharge	0		10	7		

### CT scan and image interpretation

All the study participants were scanned using a Siemens SOMATOM Emotion CT scanner in a supine position, and breath was held after inhalation. Images spanned from the apex of the lungs to the top of the diaphragm and the acquisition parameters were as follows: 120 kV; adaptive tube current (CARE Dose 4D); layer thickness, 5 mm; matrix, 512 × 512; high-resolution algorithm reconstruction; layer interval, 2 mm; and reconstruction layer thickness 1.5 mm.

All chest CT scans were subject to blind review by two radiologists (with 5 and 18 years of experience in reading chest CT) in consensus. If the results were inconsistent, a consensus was reached after negotiation. All images were viewed on both the lung (width, 1100–1300 HU; level, − 800 HU) and mediastinal (width, 300–400 HU; level, 40–50 HU) settings on the picture archiving and communication system. CT features including lesion location (two lungs, single lung, lung lobe, and lung segment), degree of lung involvement; focal (single lesion), multiple (two or more lesions, limited to 2 lung lobes), diffuse (multiple lesions, distributed in more than 2 lung lobes), lesion distribution characteristics (in the pleura, along the bronchial vascular bundle, both in pleura and along the bronchial vascular bundle), lesion morphological characteristics (ground-glass opacity, ground-glass nodules, crazy-paving pattern, air bronchogram) were recorded. These images were then evaluated using the semi-quantitative scoring method proposed by Chang et al. [12, 13] with lung involvement being determined using the separate score from each of the five lung lobes. Each lung lobe was 0–5 points, with a total possible score of 0–25 (0 point represented no involvement, 1 point less than 5% affected, 2 points 6–25% affected, 3 points 26–49% affected, 4 points 50–75% affected, 5 points more than 75% affected). The CT findings on admission, discharge, and after discharge were compared between the two groups.

### Statistical analysis

Statistical analysis was performed using SPSS software (version 23.0). Categorical variables are represented as number and frequency, while continuous variables are described using the mean, standard deviation, median, and interquartile range (IQR). The clinical and CT characteristics of the positive and negative viral RNA groups were compared using the χ ^2^ test or Fisher’s exact test. One-Way ANOVA or independent-sample t test was performed on the continuous variables based on their distribution. A *P* value of < 0.05 was considered statistically significant. Multiple comparisons were not analyzed, and given the possibility of Type I errors, these findings should be interpreted as exploratory and descriptive.

## Results

### Clinical characteristics

This case study included 42 patients with confirmed COVID-19 who were clinically cured and discharged based on the COVID-19 guidelines (Trial Version 5) [6]. The positivity rate of the anal swab samples from discharged patients was 14.3% (6/42), all of which were male (100%) (Table 1). There was no recurrence in the throat swab samples after anal swab specimens tested positive, and no close contacts were found to be infected. In the six patients with positive anal swab results two patients (33.3%) were identified through contact tracing, and four (66.7%) were identified via a family cluster. The six cases spanned all of the clinical presentations and included one mild type, three common types, and two severe types of infections. There was no significant difference in clinical type, the use of antibiotics or glucocorticoids during hospitalization between the two groups. The incidence of GI symptoms was 16.7% (7/42), 6 patients (85.7%) presenting with diarrhea were classified as common type, and one patient (14.3%) presenting with nausea and vomiting was classified as severe type. None of the six patients, positive for viral RNA on the anal swab, presented with GI symptoms. The most common symptom at onset is fever (69.0%); with other symptoms such as cough, dyspnea, myalgia, and diarrhea frequently persisting after the patient’s temperature has returned to normal. The median duration of fever in the positive group was 1 day (IQR, 0–6.3 days) which was shorter than that of the negative group (6 days [IQR, 2–9.8 days]), but the difference between the two groups was not statistically significant. The median duration of other major symptoms (except fever) in the positive patients was also shorter than that of patients with negative results (4.5 days [IQR 0–5.5 days] vs. 10.5 days [IQR 4.3–14 days], *P* = 0.022). The incidence of asymptomatic cases in the positive group was higher than that in the negative group (33.3% vs. 5.6%, *P* = 0.034) and there were no significant differences in the other clinical characteristics between the two groups (Table 2).

**Table 2:** Demographics and baseline characteristics of patients with COVID-19

	Total (*N* = 42)	Anal swab test positive (*n* = 6)	Anal swab test negative (*n* = 36)	*P* Value^a^
Sex				
Female	25(59.5%)	6(100.0%)	19(52.8%)	0.031
Male	17(40.5%)	0(0.0%)	17(47.2%)	
Age, median (IQR), y	40(30.8 ~ 46.0)	28.5(13.3 ~ 50.5)	42(32 ~ 46)	0.250
>50	9(21.4%)	1(16.7%)	8(22.2%)	0.762
≤ 50	33(78.6%)	5(83.3%)	28(77.8%)	
BMI, median (IQR)	24.4(22.2 ~ 25.7)	24.6(16.0 ~ 27.9)	24.1(22.2 ~ 25.6)	1.000
Epidemiology				
Direct exposure history	22(52.4%)	2(33.3%)	20(55.6%)	0.148
Indirect exposure history	6(14.3%)	0(0.0%)	6(16.7%)	
Family outbreak	14(33.3%)	4(66.7%)	10(27.8%)	
Incubation period, median (IQR), d	7(3 ~ 10)	9(4.5 ~ 11.3)	6.5(3 ~ 10)	0.320
Onset of symptom to, median (IQR), d				
Hospital admission	3.5(2 ~ 6)	3.0(2 ~ 5.5)	3.5(2 ~ 6)	0.697
Anal swab test	27(25 ~ 32.3)	26.5(21.5 ~ 35.3)	27(25 ~ 32.3)	0.836
Signs and symptoms				
Fever	29(69.0%)	3(50.0%)	26(72.2%)	0.281
Maximum temperature, median (IQR), °C	37.7(36.8 ~ 38.5)	37.2(36.1 ~ 38.2)	37.9(37.0 ~ 38.7)	0.235
<37·3	13(31.0%)	3(50.0%)	10(27.8%)	0.541
37·3–38	14(33.3%)	2(33.3%)	12(33.3%)	
38·1–39	8(19.0%))	0(0.0%)	8(22.2%)	
>39	7(16.7%)	1(16.7%)	6(16.7%)	
Cough	35(83.3%)	4(66.7%)	31(86.1%)	0.242
Dyspnea	3(7.1%)	1(16.7%)	2(5.6%)	0.333
Fatigue	3(7.1%)	1(16.7%)	2(5.6%)	0.333
Myalgia	5(11.9%)	0(0.0%)	5(13.9%)	0.336
Headache	4(9.5%)	0(0.0%)	4(11.1%)	0.396
Nausea and vomiting	1(2.4%)	0(0.0%)	1(2.8%)	0.683
Diarrhea	6(14.3%)	0(0.0%)	6(16.7%)	0.285
No symptoms	4(9.5%)	2(33.3%)	2(5.6%)	0.034
Comorbidities				
Diabetes	2(4.8%)	0(0.0%)	2(5.6%)	0.558
Hypertension	2(4.8%)	0(0.0%)	2(5.6%)	0.558
Hepatitis or liver cirrhosis	2(4.8%)	0(0.0%)	2(5.6%)	0.558
Chronic renal failure	1(2.4%)	0(0.0%)	1(2.8%)	0.683
Malignancy	1(2.4%)	0(0.0%)	1(2.8%)	0.683
Digestive diseases	3(7.1%)	1(16.7%)	2(5.6%)	0.333
Clinical type				
Mild type	2(4.8%)	1(16.7%)	1(2.8%)	0.701
Common type	30(71.4%)	3(50.0%)	27(75.0%)	
Severe type	7(16.7%)	2(33.3%)	5(13.9%)	
Critical type	3(7.1%)	0(0.0%)	3(8.3%)	
Mild + Common type	32(76.2%)	4(66.7%)	28(77.8%)	0.558
Severe + Critical type	10(23.8)	2(33.3%)	8(22.2%)	
Treatment				
Antibiotic	29(69.0%)	3(50.0%)	26 (72.2%)	0.281
Duration of antibiotic	6.5(0 ~ 8.3)	2.5(0 ~ 10.5)	7.0(0 ~ 8)	0.562
Glucocorticoid	29(69.0%)	4(66.7%)	25(69.4%)	0.892
Duration of glucocorticoid	4(0 ~ 5)	4.5(0 ~ 6.5)	4(0 ~ 5)	0.739
Non-Invasive Ventilation	3(7.1%)	1(16.7%)	2(5.6%)	0.333
Duration of throat swab positive, median (IQR), d	7.5(5 ~ 10.3)	10.0(7.3 ~ 12.3)	7.0(3 ~ 10)	0.208
Duration of fever, median (IQR), d	5(0.8 ~ 9.3)	1(0 ~ 6.3)	6(2 ~ 9.8)	0.120
Duration of major symptoms (except fever), median (IQR), d	8.5(3.8 ~ 13.3)	4.5(0 ~ 5.5)	10.5(4.3 ~ 14)	0.022
Hospital stays, median (IQR), d	12.5(9 ~ 18)	14.5(11 ~ 20.3)	11.5(9 ~ 17.5)	0.471

^a^ *P* values indicate differences between anal swab test positive and negtive patients. *P* < .05 was considered statistically significant

### Laboratory findings

The positive rate of the stool occult blood test (OBT) during hospitalization was 40% (2/5) in patients with positive anal swabs, and 8.7% (2/23) in the negative group (Table 3). Common causes of gastrointestinal (GI) damage in patients with positive anal swab tests were ruled out by the careful evaluation of the patients prior medical history and related examinations. The median oxygenation index of the positive anal swab group was higher than that of the negative group (486.2 mmHg [IQR 409.5–680.1 mmHg] vs. 381.0 mmHg [IQR 318.6–467.8 mmHg], *P* = 0.040). One severe-type patient had five positive throat swab samples but was negative on the anal swab test. There was no significant difference between the two groups in any of the other laboratory findings (Table 3).

**Table 3:** Laboratory Findings of Patients Infected With COVID-19 on Admission to Hospital

	Normal range	Median (IQR)			*P* Value^a^
		Total (N = 42)	Anal swab test positive (*n* = 6)	Anal swab test positive (*n* = 36)	
Leukocyte count, × 109/L	3.5 ~ 9.5	5.2(3.9 ~ 6.2)	5.0(3.6 ~ 6.6)	5.3(4.1 ~ 6.3)	0.820
Lymphocyte count, ×109/L	0.8 ~ 4	1.3(0.9 ~ 1.8)	1.8(0.6 ~ 2.3)	1.2(1.0 ~ 1.6)	0.539
lymphocyte percentage, %	20 ~ 40	26.9(20.4 ~ 33.1)	33.4(17.6 ~ 36.0)	26.2(20.1 ~ 32.7)	0.408
Haemoglobin, g/L	130 ~ 175	142.5(131.0 ~ 155.5)	147(134.3 ~ 160.0)	139(131.0 ~ 154.8)	0.516
Platelet count, ×109/L	125 ~ 350	181(145.8 ~ 231.0)	234.5(127.5 ~ 305.3)	179.5(146.3 ~ 223.8)	0.493
ESR, mm/1 h	0 ~ 15	25(11.3 ~ 44.8)	18(7.1 ~ 36.0)	26(12.8 ~ 45.3)	0.210
C-reactive protein, mg/L	0 ~ 6	11.3(2.4 ~ 35.2)	9.3(1.7 ~ 33.3)	11.3(2.4 ~ 36.6)	0.471
Procalcitonin, μg/L	0.0 ~ 0.5	0.05(0.03 ~ 0.05)	0.05(0.03 ~ 0.05)	0.05(0.03 ~ 0.05)	0.661
Total bilirubin, μmol/L	3.42 ~ 8.2	7.2(4.7 ~ 8.7)	6.5(2.8 ~ 7.5)	7.5(4.8 ~ 8.9)	0.159
Alanine aminotransferase, U/L	0 ~ 40	25.5(14.0 ~ 40.0)	30(7.3 ~ 57.5)	24(15.0 ~ 37.3)	0.793
Aspartate aminotransferase, U/L	5 ~ 49	27.5(22.0 ~ 35.5)	27.5(22.0 ~ 43.8)	27.5(22.0 ~ 35.0)	0.739
Albumin, g/L	60 ~ 85	43.7(40.6 ~ 45.9)	46.1(43.2 ~ 48.2)	42.7(40.5 ~ 45.5)	0.075
Creatine kinase, U/L	26 ~ 196	91.5(68.8 ~ 133.3)	108.5(85.5 ~ 195.5)	90(61.3 ~ 126.3)	0.281
Lactate dehydrogenase, U/L	109 ~ 245	244.5(202.5 ~ 308.5)	240(193 ~ 290)	244.5(202.8 ~ 309.5)	0.586
Creatinine, μmol/L	59 ~ 104	61.0(51.5 ~ 70.9)	60.9(52.0 ~ 64.5)	62.7(51.2 ~ 71.2)	0.713
Blood urea nitrogen, mmol/L	1.43 ~ 8.2	3.3(2.5 ~ 5.0)	4.7(4.0 ~ 5.3)	3.1(1.5 ~ 7.7)	0.040
Glucose, mmol/L	3.89 ~ 6.11	5.6(5.2 ~ 6.8)	5.0(4.7 ~ 6.6)	5.7(5.3 ~ 6.9)	0.123
D-dimers, mg/L	0 ~ 0.55	0.3(0.2 ~ 0.4)	0.3(0.2 ~ 0.5)	0.3(0.2 ~ 0.4)	1.000
Prothrombin time, s	9 ~ 14	11.4(11.0 ~ 11.9)	11.0(10.8 ~ 12.9)	11.4(11.0 ~ 11.9)	0.586
APTT, s	20 ~ 40	26.1(23.7 ~ 28.6)	26.2(23.1 ~ 30.6)	26.1(24.5 ~ 27.6)	0.958
Thrombin time, s	14 ~ 21	16.4(15.8 ~ 17.6)	16.9(15.8 ~ 18.0)	16.3(15.8 ~ 17.5)	0.562
Fibrinogen, g/L	2 ~ 4	3.4(2.9 ~ 4.5)	3.1(2.4 ~ 4.5)	3.5(3.1 ~ 4.5)	0.314
Endotoxin, pg/mL	<10	90.4(8.3 ~ 176.0)	31.4(4.4 ~ 388.9)	100.7(8.5 ~ 176.0)	0.576
SpO2, %	91.9 ~ 99.9	98(97 ~ 99)	98(98 ~ 100)	98(97 ~ 99)	0.265
PaCO2, mmHg	35 ~ 45	37.0(34 ~ 41)	36.0(33.4 ~ 42.7)	37.0(34.1 ~ 41.0)	0.933
PaO2:FIO2, mmHg	400 ~ 500	388.1(330.2 ~ 487.1)	486.2(409.5 ~ 680.1)	381.0(318.6 ~ 467.8)	0.040
BNP, ng/L	0 ~ 125	50(50 ~ 58.3)	50(50 ~ 50)	50(50 ~ 82.3)	0.600
Troponin I, μg/L	0 ~ 1.68	0.8(0.6 ~ 0.9)	0.8(0.6 ~ 0.9)	0.8(0.6 ~ 0.9)	0.741
Fecal occult blood test, positive		4(14.3%)	2(40%)	2(8.7%)	0.135

^a^*P* values indicate differences between anal swab test positive and negtive patients. *P* < .05 was considered statistically significant. *ESR* erythrocyte sedimentation rate, *APTT* Activated partial thromboplastin time

### CT findings

**Fig. 1:**
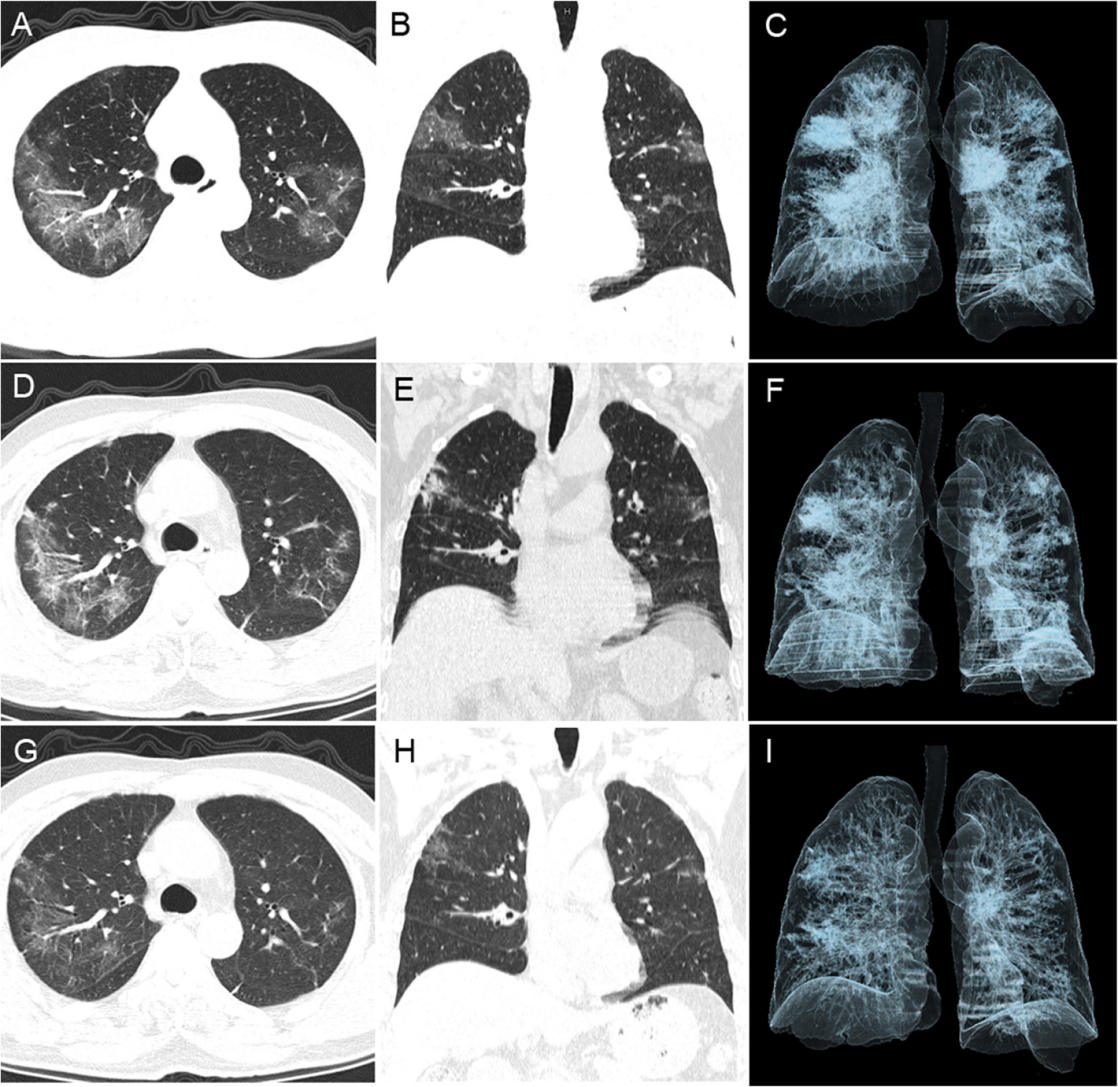
Patient 3 had a positive anal swab sample after discharge. This aged male, who was admitted to the hospital because of a cough and dyspnea, presented without fever (body temperature 36.1 °C) or diarrhea. His OBT was positive and his throat swab RNA tests were positive for 13 days. His chest CT, taken on admission (February 10, 2020), showed bilateral multiple GGO and intralobular interstitial thickening and peripheral distribution (CT score = 17) (A-C). His CT on discharge (February 20, 2020) showed absorption of multiple GGO, but consolidation and aggravated linear opacities, the lesions absorption was slow (CT score = 14) (D-E). CT after discharge (March 8, 2020) showed continuous absorption of multiple lesions, with a small amount of remaining GGO and linear opacities (CT score = 10) (G-I)

**Fig. 2:**
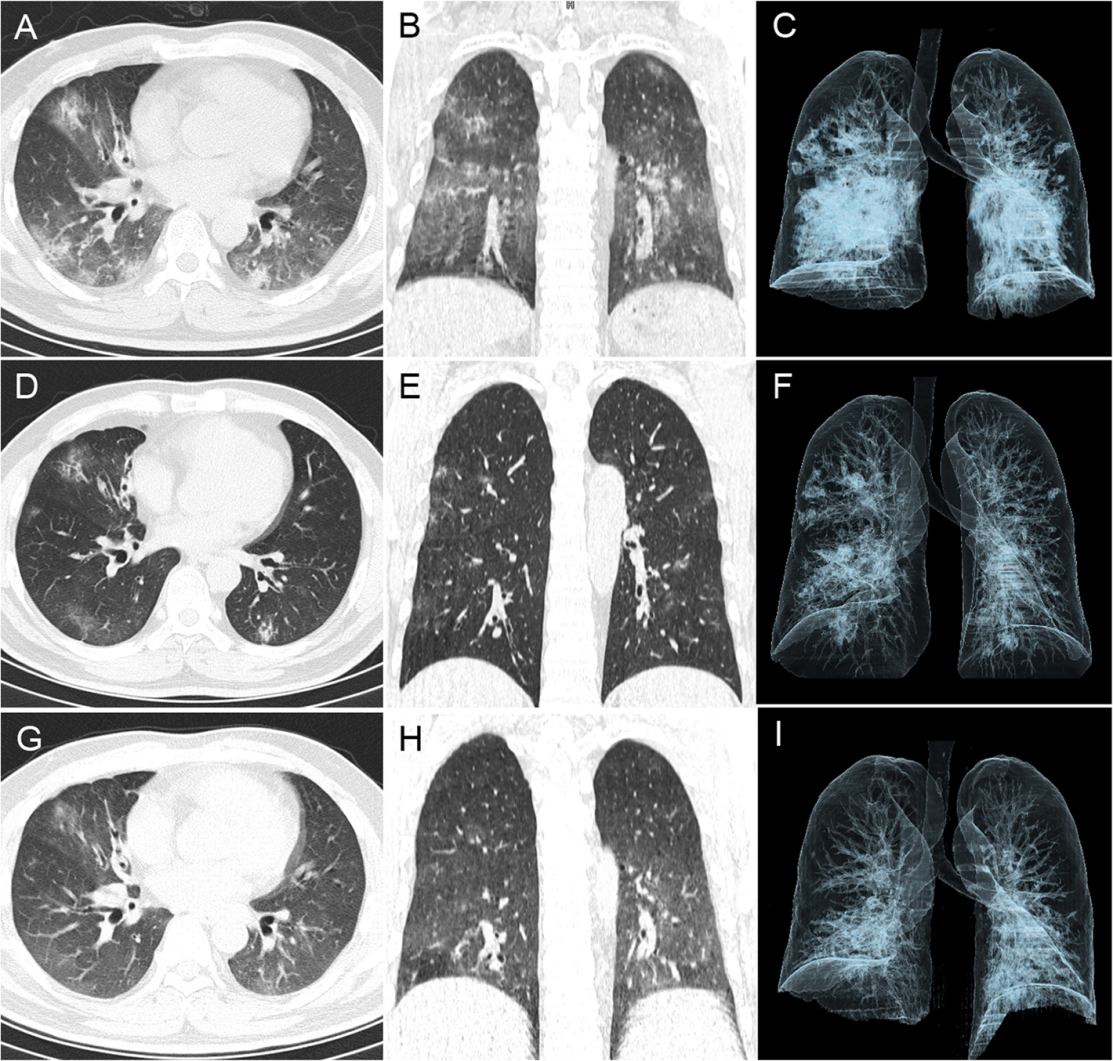
Patient 4 had a positive anal swab after discharge. This middle-aged male was admitted to the hospital because of fever (maximum body temperature 39.5 °C), cough, and dyspnea. His OBT was positive and he had positive throat swab samples for 12 days. The chest CT on admission (January 20, 2020) showed markedly reduced lung translucency, bilateral multiple GGO and consolidations and peripheral distribution (CT score = 13) (A-C). CT on discharge (February 10, 2020) showed lung transparency returned to normal and absorption of multiple GGO, but some irregular consolidations remained (CT score = 10) (D-E). CT after discharge (February 24, 2020) showed continuous absorption of multiple lesions, with only a small amount of GGO remaining (CT score = 7) (G-I)

**Fig. 3:**
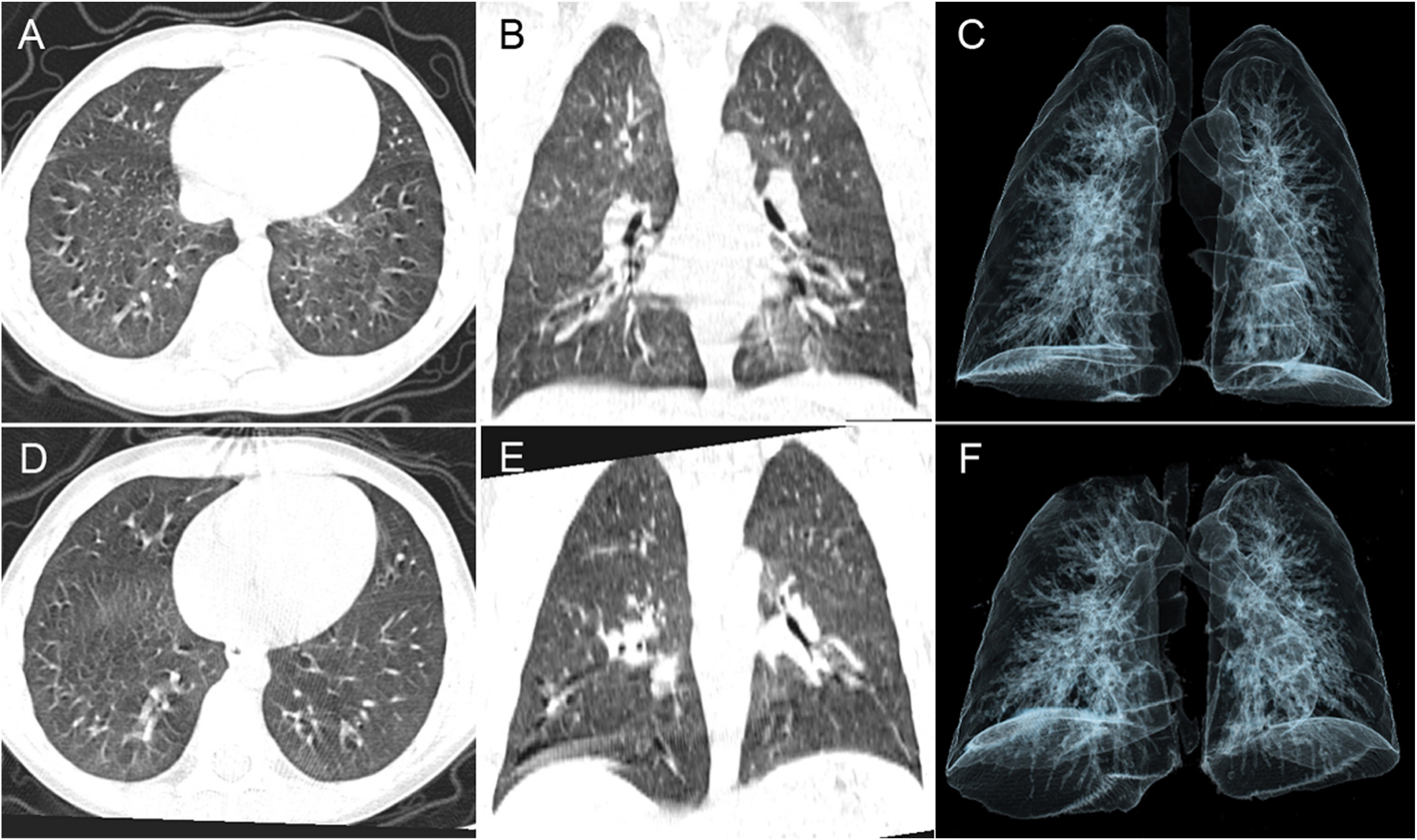
Patient 2 had a positive anal swab for SARS-CoV-2 after discharge. The young male, who was admitted to the hospital because of persistent cough (5 days) and presented without fever (body temperature 36.0 °C) or diarrhea. His OBT was negative, and his throat swabs were positive for only 9 days. The chest CT administered on admission (February 10, 2020) showed a focal GGO in the left lower lobe and peripheral distribution (CT score = 1) (A-C). The CT on discharge (February 20, 2020) showed complete absorption of this lesion (CT score = 0) (D-E)

**Fig. 4:**
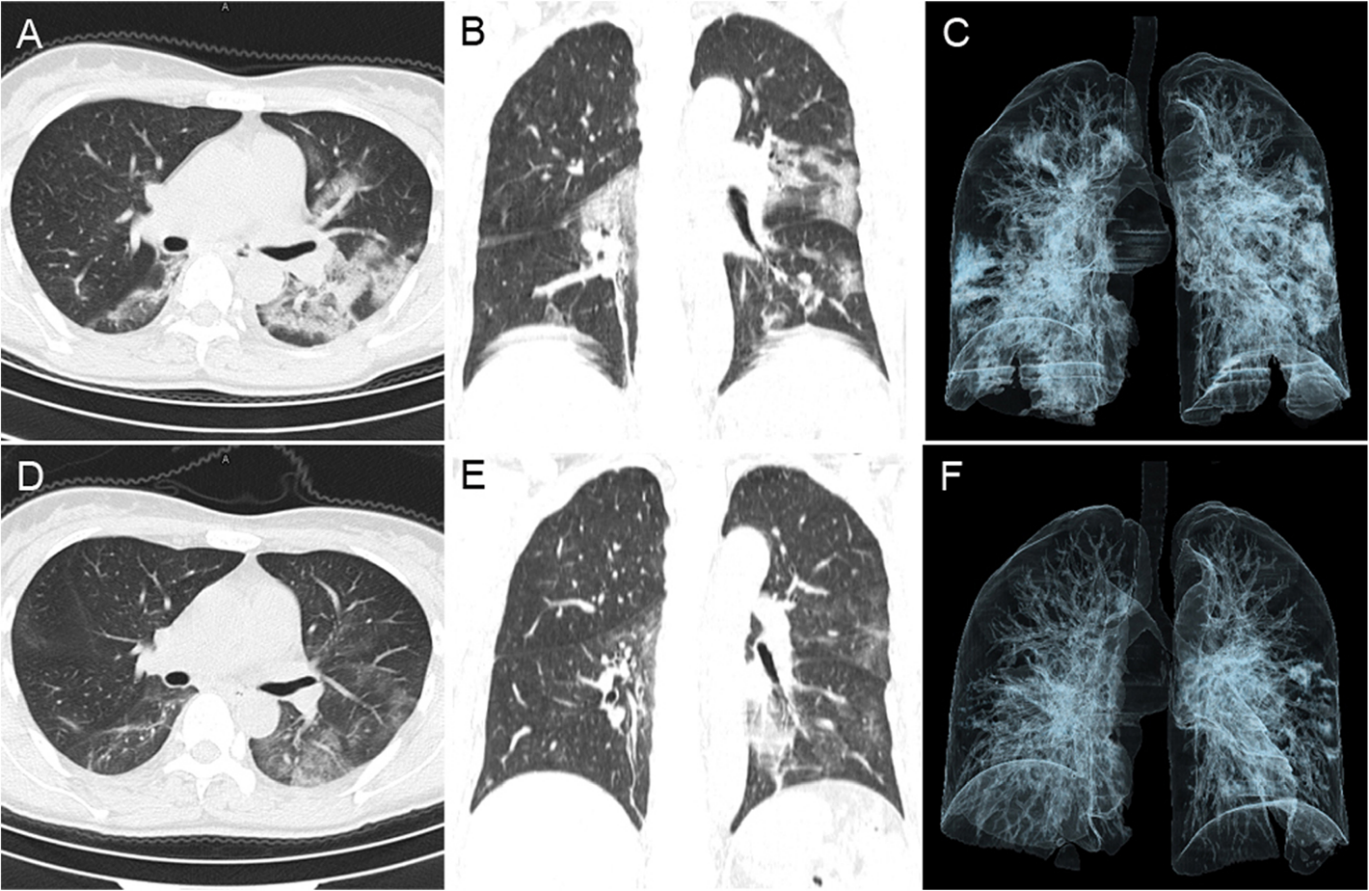
A patient with an anal swab negative for SARS-CoV-2 after discharge. This middle-aged female was admitted to the hospital because of a persistent cough (6 days) and fever (maximum body temperature of 39.6 °C), without diarrhea. Her OBT was negative, and she had positive throat swabs for only 6 days. The chest CT on admission (February 6, 2020) showed bilateral multiple GGO and consolidation, central and peripheral distribution (CT score = 11) (A-C). CT on discharge (February 12, 2020) showed significant absorption of these bilateral lung lesions (CT score = 5) (D-E)

For the patients in the positive group: Patient 1 did not show any abnormalities on CT. Abnormal CT findings were found in patients 2, 3, and 4 on admission (Figs. 1, 2 and 3). Patient 2 was a child with a focal GGO in the left lower lobe and a CT score of 1. Two severe-type patients (patients 3 and 4) presented with multiple GGO and consolidations in both lungs. CT scores on admission, discharge, and after discharge for Patient 3 were 17, 14, and 10, respectively, and the corresponding CT scores for Patient 4 were 13, 10, and 7, respectively. There were no significant differences in the number of segments involved, distribution of lesions, imaging features, or CT score on admission between the two groups. The absorption on the CT scans of patients 3 and 4 was slow (Figs. 1, 2, 3 and 4), but there were no significant difference in the CT scores at discharge between the two groups. The CT scores of the positive group were generally higher than those of the negative group after discharge (8.5 ± 2.1 vs. 3.8 ± 2.2, *P* = 0.038) (Table 4).

**Table 4:** Imaging characteristics of patients with COVID-19

	Total (*N* = 31)	Positive (n = 4)	Negative (*n* = 27)	*P* Value ^a^
First CT, negative, (%)	3(9.7%)	1(25.0%)	2(7.4%)	0.349
First CT, positive, (%)	28(90.3%)	3(75.0%)	25(92.6%)	
Number of involved segments	9.3 ± 6.1	11 ± 8.9	9.1 ± 5.9	0.726
Involvement, (%)				
Single lobe	5(17.9%)	1(33.3%)	4(16.0%)	0.459
Bilateral lobe	23(82.1%)	2(66.7%)	21(84.0%)	
Distribution, (%)				
Central	0	0	0	–
Peripheral	15(53.6%)	1(33.3%)	14(56.0%)	0.583
Central + Peripheral	13(46.4%)	2(66.7%)	11(44.0%)	
Degree, (%)				
Single lesion	4(14.3%)	1(33.3%)	3(12.0%)	0.217
Multiple lesions	13(46.4%)	0(0.0%)	13(52.0%)	
Diffuse lesions	11(39.3%)	2(66.7%)	9(36.0%)	
Imaging Features, (%)				
GGO	27(96.4%)	1(66.7%)	25(100.0%)	0.107
Consolidation	19(67.9%)	3(100.0%)	16(64.0%)	0.530
Linear opacities	19(67.9%)	2(66.7%)	17(68.0%)	1.000
Mixed type	22(78.6%)	2(66.7%)	20(80.0%)	0.530
Interstitial change				
Interlobular septal thickening	3(10.7%)	1(3.3%)	2(8.0%)	0.298
Intralobular interstitial thickening	21(75.0%)	2(66.7%)	19(76.0%)	1.000
Other signs, (%)				
Nodule	8(28.6%)	1(33.3%)	7(28.0%)	1.000
Dilatation of vessels	26(92.9%)	2(66.7%)	24(96.0%)	0.206
Bronchial wall thickening	13(46.4%)	2(66.7%)	11(44.0%)	0.583
Air bronchogram	5(17.9%)	0(0.0%)	5(20.0%)	1.000
Crazy-paving pattern	3(10.7%)	0(0.0%)	3(12.0%)	1.000
Halo sign	7(25.0%)	1(33.3%)	6(24.0%)	1.000
Reversed halo sign	0(0.0%)	0(0.0%)	0(0.0%)	–
Pleural thickening	4(14.3%)	0(0.0%)	4(16.0%)	1.000
Hydrothorax	0(0.0%)	0(0.0%)	0(0.0%)	–
Lymphadenectasis	0(0.0%)	0(0.0%)	0(0.0%)	–
CT scores				
On admission	7.7 ± 4.6	10.3 ± 8.3	7.4 ± 4.1	0.532
On discharge	5.6 ± 3.5	8.0 ± 7.2	5.3 ± 2.9	0.433
After discharge	4.4 ± 2.7	8.5 ± 2.1	3.8 ± 2.2	0.038

^a^*P* values indicate differences between anal swab test positive and negative patients. *P* < .05 was considered statistically significant

## Discussion

Since COVID-19 mainly affects the respiratory system [14, 15], anal swab and stool sample testing for SARS-CoV-2 RNA has only recently gained attention. Some patients without GI symptoms may present with positive stool samples [3, 7], and a positive SARS-CoV-2 RNA result from an anal swab or stool specimen does not necessarily indicate that there must be live virus in the patient’s stool since the viral particles trapped in the respiratory secretions could be swallowed and processed through the digestive tract. Several case reports have shown that live virus can be detected in stool specimens from COVID-19 patients [16–20]. However, it is still uncertain whether SARS-CoV-2 in feces might be an additional source of transmission. No previous studies have investigated the clinical or imaging characteristics of patients with positive anal swab or stool specimens.

In the current case series, six patients (14.3%) were found to have positive anal swab samples after discharge. The positivity rate for SARS-CoV-2 RNA in feces samples from hospitalized patients with COVID-19 was reported to be between 29 and 53% [19, 20]. The reason why our positive rate was lower than theirs may be that they tested stool specimens from hospitalized patients at earlier time points, while we tested anal swabs from patients who met the discharge criteria and thus represent a later time point. Xiao et al. [18] tested serial stool specimens from a critically ill patient and found that there were only RNA fragments and not infectious viral particles in the feces collected at later time points. In the anal swab positive group, 40% of patients (2/5) tested positive for OBT, while only 8.7% (2/23) were OBT positive in the negative group. There was no diarrhea in the anal swab positive group, but six patients in the negative group reported GI symptoms. Diarrhea does not necessarily correlate with OBT, so we speculate that diarrhea may be only a premonitory symptom of SARS-CoV-2 infection of the GI tract. The higher positive rate of OBT during hospitalization in patients with positive anal swab samples suggests that a positive OBT may be a sign that SARS-CoV-2 is damaging the GI tract. OBT-positive patients were positive for viral RNA on the anal swab samples for longer. This suggests that OBT may be a better indicator of GI SARS-CoV-2 infection and damage than symptomology. The mechanism by which SARS-CoV-2 causes GI damage is still unclear. In humans and other mammals, coronaviruses mainly target the upper respiratory tract, GI tract, and central nervous system [16, 21]. SARS-CoV-2 belongs to the same family as the SARS-CoV that caused the SARS outbreak in 2003. It is currently known that in addition to damaging the respiratory tract, SARS-CoV also damages the intestine. The main driver for this damage is the fact that cell receptor-angiotensin-converting enzyme II (ACE2) is expressed in both the human respiratory tract and the esophagus, small intestine, and colon [21–23]. Some studies indicate that 3–10% of patients with COVID-19 develop GI symptoms [14, 15]. We can thus hypothesize that SARS-CoV-2 is transmitted through ACE2 receptors in the GI tract damaging the intestine, leading to hemorrhage, which could be detected by OBT.

Our study found that the median duration of the major symptoms was shorter in those patients with positive anal swabs than in those without. The reason for the less severe symptoms and faster recovery in the positive group remains unclear, but may indicate that these patients reached the recovery phase of the disease faster. We also found that the anal swabs tests started to come back negative for viral RNA at around 7 days, with this being extended to 9 days in more severe cases. This observation suggests that the virus may exist in severe patients for longer. Live SARS-CoV-2 was isolated from feces [16–20], indicating that fecal-oral or fecal-respiratory transmission is possible via aerosolized feces. During the SARS epidemic, a large-scale community outbreak in Hong Kong, China, was caused by patient excreta eventually resulting in 321 infections and 42 deaths [24]. This indicates that the fecal excreta of patients with SARS can be infectious. Although the infectivity of patients with positive anal swab viral RNA is still uncertain, we recommend that anal swab or fecal samples are tested for viral RNA and that OBT is evaluated at diagnosis and over the course of treatment in cases of COVID-19 to minimize the risk of fecal transmission. Given this discharge and hospital cleaning practices should pay more attention to those who have a positive OBT result. However, this recommendation still needs to be validated in a larger clinical cohort.

This study has several limitations. First, this was a retrospective study. Second, our cohort size was small since only 42 discharged patients had any anal samples collected and the positive rate of the anal swab tests was also very low, thus the findings of this study should be interpreted as exploratory and descriptive. Third, we lack more detailed clinical information, including the absence of patient sera to evaluate viremia. We could not compare the viral loads of respiratory and digestive tract specimens during hospitalization. Fourth, false negatives could potentially interfere with our results. Lastly, the infectivity of patients with positive stool samples cannot be established. We hope that the preliminary results of this study will stimulate further research in this area.

## Conclusion

In conclusion, 14.3% of the discharged patients in this study were found to be positive for SARS-CoV-2 RNA in anal swab samples. They did not have GI symptoms and had similar CT features as the negative patients, and their main symptoms disappeared early, which may make these patients easier to ignore. A positive OBT may indicate GI damage caused by SARS-CoV-2. Further follow-up and research are needed.

## Data Availability

The datasets used and/or analyzed during this study are available from the corresponding author (Weihua Liao, owenliao@csu.edu.cn) upon reasonable request.
